# Post treatment imaging in patients with local advanced cervical carcinoma

**DOI:** 10.3389/fonc.2022.1003930

**Published:** 2022-10-27

**Authors:** S. Ciulla, V. Celli, A. A. Aiello, S. Gigli, R. Ninkova, V. Miceli, G. Ercolani, M. Dolciami, P. Ricci, I. Palaia, C. Catalano, L. Manganaro

**Affiliations:** ^1^ Department of Radiological, Oncological and Pathological Sciences, Sapienza, University of Rome, Rome, Italy; ^2^ Department of Medical Sciences, University of Cagliari, Cagliari, Italy; ^3^ Department of Maternal and Child Health and Urological Sciences, Sapienza, University of Rome, Rome, Italy

**Keywords:** MRI, gynecologic malignancies, oncology, cervical cancer, gynecology

## Abstract

Cervical cancer (CC) is the fourth leading cause of death in women worldwide and despite the introduction of screening programs about 30% of patients presents advanced disease at diagnosis and 30-50% of them relapse in the first 5-years after treatment. According to FIGO staging system 2018, stage IB3-IVA are classified as locally advanced cervical cancer (LACC); its correct therapeutic choice remains still controversial and includes neoadjuvant chemo-radiotherapy, external beam radiotherapy, brachytherapy, hysterectomy or a combination of these modalities. In this review we focus on the most appropriated therapeutic options for LACC and imaging protocols used for its correct follow-up. We explore the imaging findings after radiotherapy and surgery and discuss the role of imaging in evaluating the response rate to treatment, selecting patients for salvage surgery and evaluating recurrence of disease. We also introduce and evaluate the advances of the emerging imaging techniques mainly represented by spectroscopy, PET-MRI, and radiomics which have improved diagnostic accuracy and are approaching to future direction.

## 1 Introduction

Cervical cancer (CC) is the fourth leading cause of death in women worldwide, with an estimated global incidence of 470,000 new cases per-year (1).

Despite the introduction of screening programs about 30% of patients presents advanced disease at diagnosis and 30-50% of them relapse in the first 5-years after treatment (2).

Accurate staging is crucial to select a tailored treatment. According to the new International federation of gynecology and obstetrics (FIGO) staging system 2018, stage IA, IB1, IIa1 are classified as early stage of disease and can be treated with surgery, either fertility sparing trachelectomy or radical surgery while stages IB3-IVA are classified as locally advanced cervical cancer (LACC) (**Supplementary Table I**) (3). For this group of patients surgery remains still controversial, and options are neoadjuvant chemo-radiotherapy, external beam radiotherapy, brachytherapy, hysterectomy or a combination of these modalities (4–6).

Imaging plays a key role in therapeutic strategy allowing selection of responding and non-responding patients after treatment, early determination of additional surgical salvage if needed in presence of residual tumor after radiotherapy and detection of tumor recurrence during post treatment follow-up (7–9).

In this review we focus on the main therapeutic options in patients with LACC and on the wide spectrum of imaging findings after radiotherapy and surgery; moreover, we discuss the role of imaging in evaluating treatment response and selecting patients for salvage surgery in presence of residual tumor after radio-chemotherapy.

## 2 Research method

The literature search included articles published between 2002 and 2022 from MEDLINE, Embase, and the Cochrane Library. The following MeSH keywords were matched to guide the literature search on Pubmed: (LACC) AND (MRI) AND ((treatment) OR (follow-up)) 82; (LACC) AND (MRI) AND (recurrence) 16; (LACC) AND (MRI) AND (complication) 14; (cervical cancer) AND (MRI) AND (radiomics) 75; (cervical cancer) AND (MRI) AND (spectroscopy) 61; (cervical cancer) AND (MRI) OR (PET-MRI) 74. We included articles that provided detailed information on imaging modalities, treatment, follow-up, and recurrence of LACC, excluding those that did not properly fulfill the goal of our review. Next, case reports, case series, and articles providing views and opinions were excluded.

Our initial literature search included approximately 322 articles; 227 articles were excluded based on the previous criteria. 95 articles were selected for this review.

## 3 LACC treatment

Nowadays, the treatment of choice for LACC is concomitant chemoradiation therapy (CCRT). However, in case of disease persistence after CCRT, some authors suggest switching to salvage surgery although there is no shared consensus on this. In addition, some authors support the advantage of NACT plus RS as viable alternative treatment.

### 3.1 Concurrent chemoradiotherapy

Concurrent chemoradiation therapy (CCRT) which generally consists of cisplatin-based chemotherapy and external-beam radiotherapy followed by brachytherapy is the standard organ-preservation treatment for LACC and has become a cornerstone of treatment (4).

CCRT is the optimal choice for stages IB3, II, III and IVA of the disease improving local control and reducing the risk of local regional recurrence in comparison with radiation therapy alone. CCRT provides active systemic cytotoxic agents against CC with the potential to enhance tumor radiosensitivity and to eradicate micro-metastasis.

CCRT allows to decrease of 30% to 50% the risk of death compared to radiotherapy (RT) alone in accordance to the Meta-analysis Group of Medical Research Council Clinical Trials Unit of London which affirmed that chemoradiotherapy leads to a 6% improvement in 5-year survival (HR, 0.81; P<.001) (10). Datta et al. performed another meta-analysis in 2017 based on 2445 patients with > 95% squamous cell carcinoma (SCC) histology receiving either CCRT or RT only without surgery. The results confirmed that CCRT significantly improves outcomes, with increased of local control rate (LCR) and overall survival (OS) rates of 8.4% (p < 0.001) and 7.5% (p < 0.001), respectively (11).

#### 3.1.1 CCRT followed by surgery

The role of completion surgery after CCRT is currently controversial, since surgery has a high postoperative morbidity (12, 13). The rate of residual disease after CCRT is 40%, and these patients generally have a poor prognosis because they show scarce response to cisplatin-based chemotherapy (14). In these cases, some authors propose radical hysterectomy as an adjunctive treatment, although no guidelines recommend it as a treatment for residual tumors.

Recent literature affirms different results regarding the role and benefit of surgery after CCRT.

Some authors supported the positive impact of adjuvant surgery after CCRT: Lèguevaque et al. argued that completion surgery could improve disease-free survival (DFS), in agreement with Yoshida et al. who also obtained more favorable survival results after adjuvant surgery (15, 16).

On the other hand, Fanfani et al. and Cochrane et al. observed no significant differences in DFS and OS (17, 18).According to Kim G. Van Kol, salvage surgery should be performed only if residual disease is histologically confirmed by biopsy in patients treated with CCRT, to avoid unnecessary surgery and complications (14). Surgery after CCRT undoubtedly leads to improved local control rates; however, distant recurrence often occurs in LACC (19). Further prospective randomized trials should be conducted to evaluate the survival benefit of this strategy.

### 3.2 NACT followed by RS

Neoadjuvant chemotherapy (NACT) followed by radical surgery (RS) is considered as a valid alternative for LACC and is currently used in many countries (20, 21). Several advantages have been suggested for NACT plus RS: tumor size reduction and possibility of surgical resection, systemic action and consequent loco-regional and distant disease control, sterilization of micrometastasis (22, 23).

According to Benedetti Panici, RS after NACT is a feasible option in LACC with an acceptable survival outcome and mild surgical complications with marginal impact on quality of life (24). Gupta et al. analyzed 635 patients with stages IB2, IIA and IIB and compared NACT followed by surgery with platinum-based CCRT. The authors found that 5-year DFS was lower (69.3% vs 76.7%; HR 1.38, 95% CI 1.02-1.87, p = 0.038) in the NACT group followed by surgery with no significant difference in 5-year OS (75.4% vs 74.7%; HR 1.02, 95% CI 0.75-1.40, p = 0.87) (25). More recently, Zhao et al., on 2158 patients, demonstrated no differences in terms of OS, Progression-Free Survival (PFS), local or distant recurrences (26) (**Supplementary Table II**).

## 4 Imaging algorithm and follow-up

In patients with LACC a pre-treatment MRI (Magnetic Resonance Imaging) is performed for loco-regional staging. Mid-treatment MRI (after 5 weeks of concurrent cisplatin chemotherapy with external beam radiation therapy (EBRT) and before intra-cavitary brachytherapy) allows brachytherapy dose-adjustment in proportion to the residual tumour volume (4). This increases local tumour control, reduces toxicity and improves survival. Choose proper sequences and correct plane angles is extremely important to avoid pitfall in local staging of CC. The central role in anatomic assessment of pelvic structures is assigned to T2-weighted imaging (T2WI); T2 sequences should be acquired with thin section (3-4 mm) and field of view (FOV) of 20-24 mm to provide high anatomic resolution and acquired on the cervical axis to provide better locoregional staging (27). In fact, for patients undergoing chemo-radiotherapy treatments, without hysterectomy, T2 sequences oriented on cervical axial and coronal planes are strongly recommended; these are acquired along planes perpendicular and parallel to the endocervical axis (para-axial and para-coronal plane). According to the European Society of Urogenital Radiology (ESUR) guidelines, at least one para-axial oriented plane is required for diffusion weighted images (DWI) (**Supplementary Table III**) (28).

Conversely, after complementary surgery no more angled planes are required and all the sequences are acquired on sagittal, coronal and axial pure plane. Axial/coronal T2WI or T1-weighted imaging (T1WI) from the renal hilum to the groin are suggested to assess the presence of hydronephrosis and bone and lymph node metastases. Moreover, fluorodeoxyglucose-positron emission tomography (FDG-PET) scanned during radiotherapy treatment may facilitate tailored radiation (e.g. adjustment of EBRT field in relation to the para-aortic lymph node (LN) status) and, if standardized, potentially predict outcome. According to National Comprehensive Cancer Network (NCCN) guidelines, CC follow-up/surveillance changes according to FIGO stage and MRI is considered the preferred imaging modality for assessing locoregional tumor extension while FDG-PET/Computed Tomography (CT) is indicated for nodal and distant staging (4).

Follow-up protocols for LACC include: for stage IB3 or patients who required post-operative adjuvant radiation or chemoradiation a whole body PET/TC FDG usually performed at 3-6 months after completion of CCRT; for stage II-IVa a whole body PET/CT (preferred) or chest/abdomen/pelvic CT with contrast within 3-6 months of completion of therapy, moreover a MRI with contrast is to be considered 3-6 months after the end of treatment. In all cases, the choice and addition of imaging techniques should be evaluated based on symptomatology or clinical concern for recurrence and PET/CT and MRI are considered the techniques of choice (4).

### 4.1 MRI

MRI is now widely accepted as the most effective modality for detection, staging, treatment planning and follow-up of CC; on MRI, the tumor presents an intermediate signal intensity (SI) on T2WI, a high signal intensity (SI) on DWI at high b-value and a low SI on the apparent diffusion coefficient (ADC) map (29).

**Figure 1:**
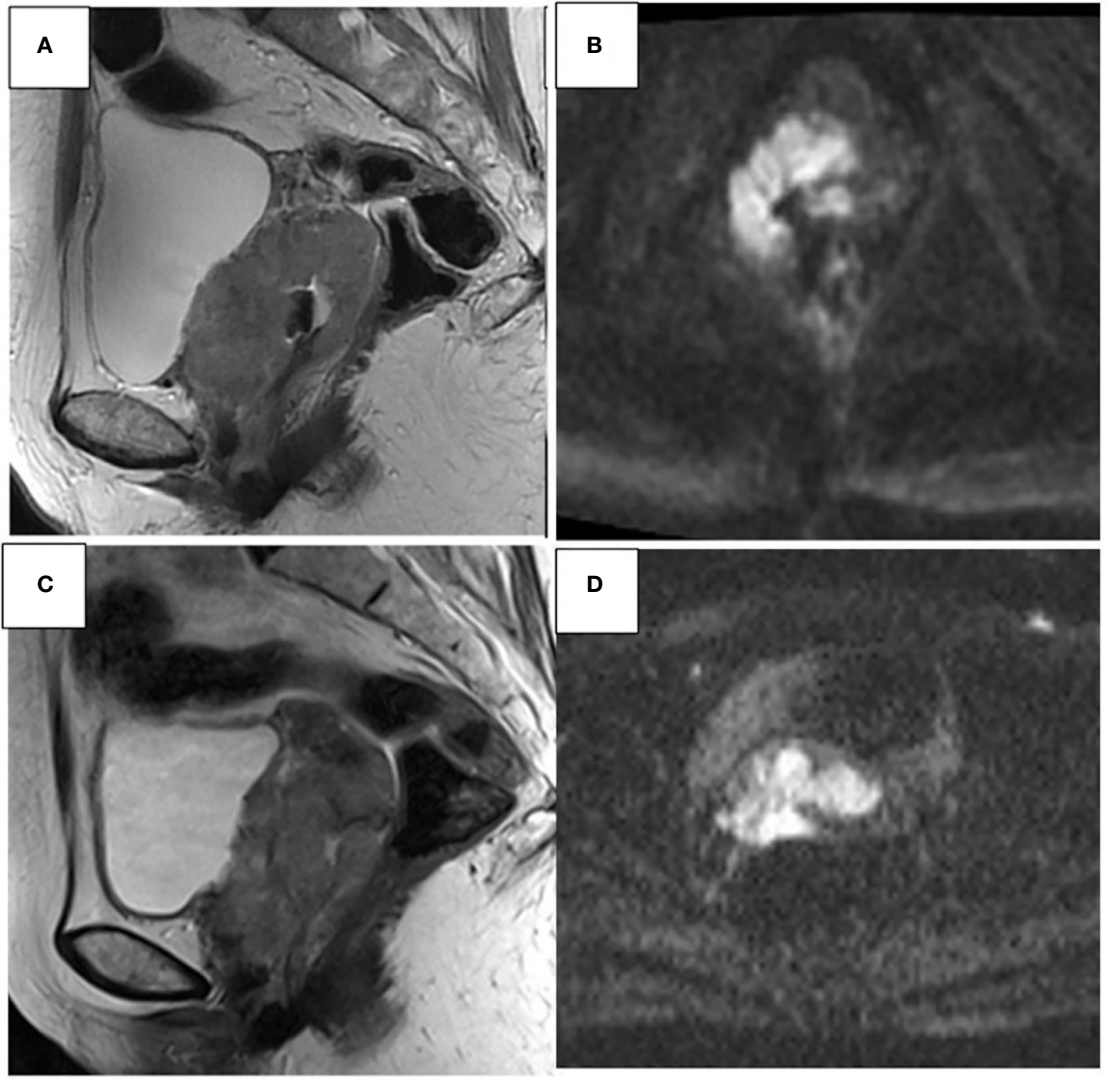
45- year-old woman. Sagittal T2W and axial DWI MR images show an invasive CC with parametrial invasion and extension of the upper 2/3 of the vagina **(A, B)**. Stable disease **(C, D)** after CCRT, CC is changed in morphology and size; however, infiltration of the vaginal fornix, upper 2/3 of the vagina, and both parameters remain.

**Figure 2:**
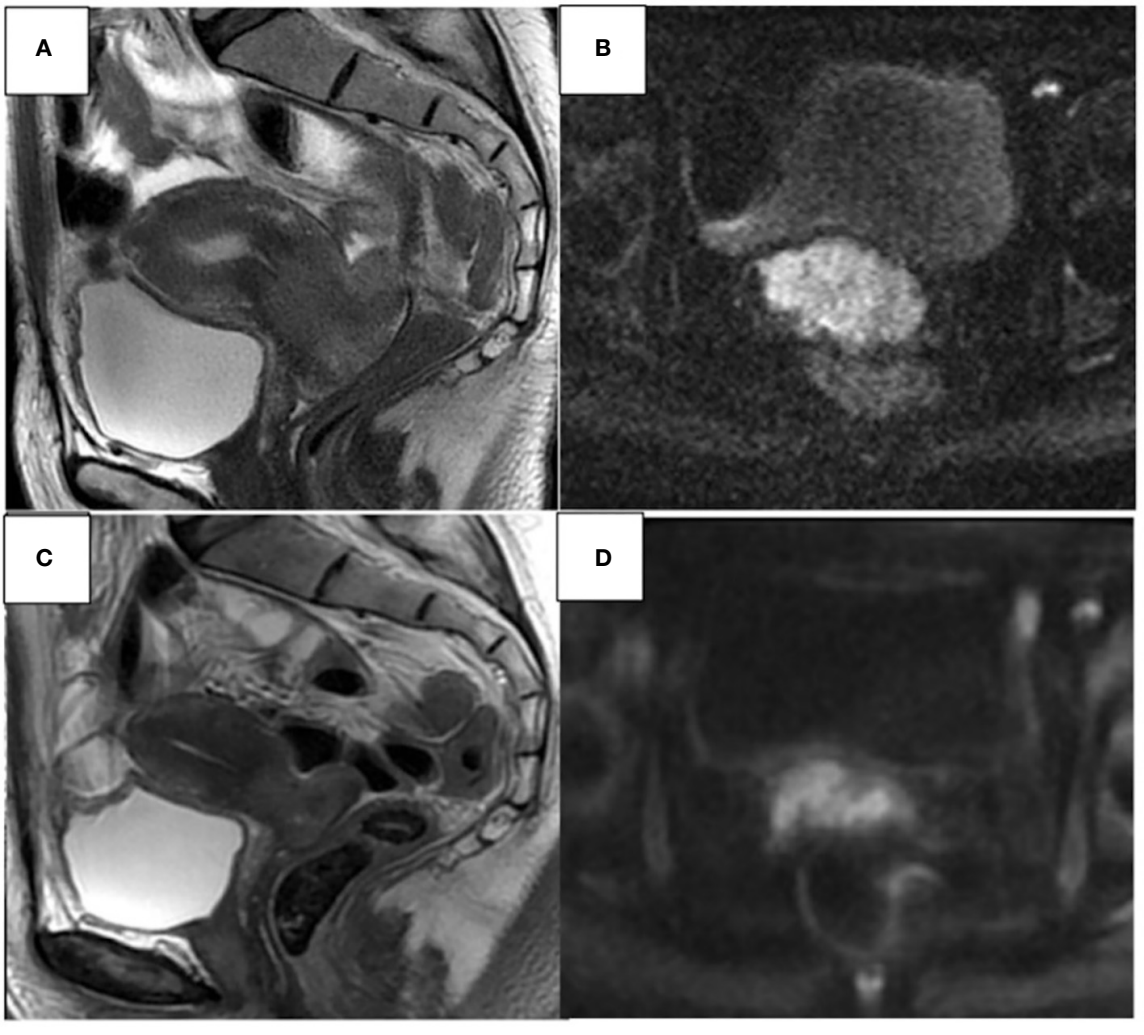
48- year-old woman. Sagittal T2W and axial DWI MR images show an invasive CC extending from the uterine isthmus to the external uterine orifice, laterally infiltrating both vaginal fornices, and extending beyond the stromal ring with extensive infiltration of the parameters. **(A, B)**. Partial response **(C, D)** approximately 30% reduction of cervical heteroplastic tissue.

**Figure 3:**
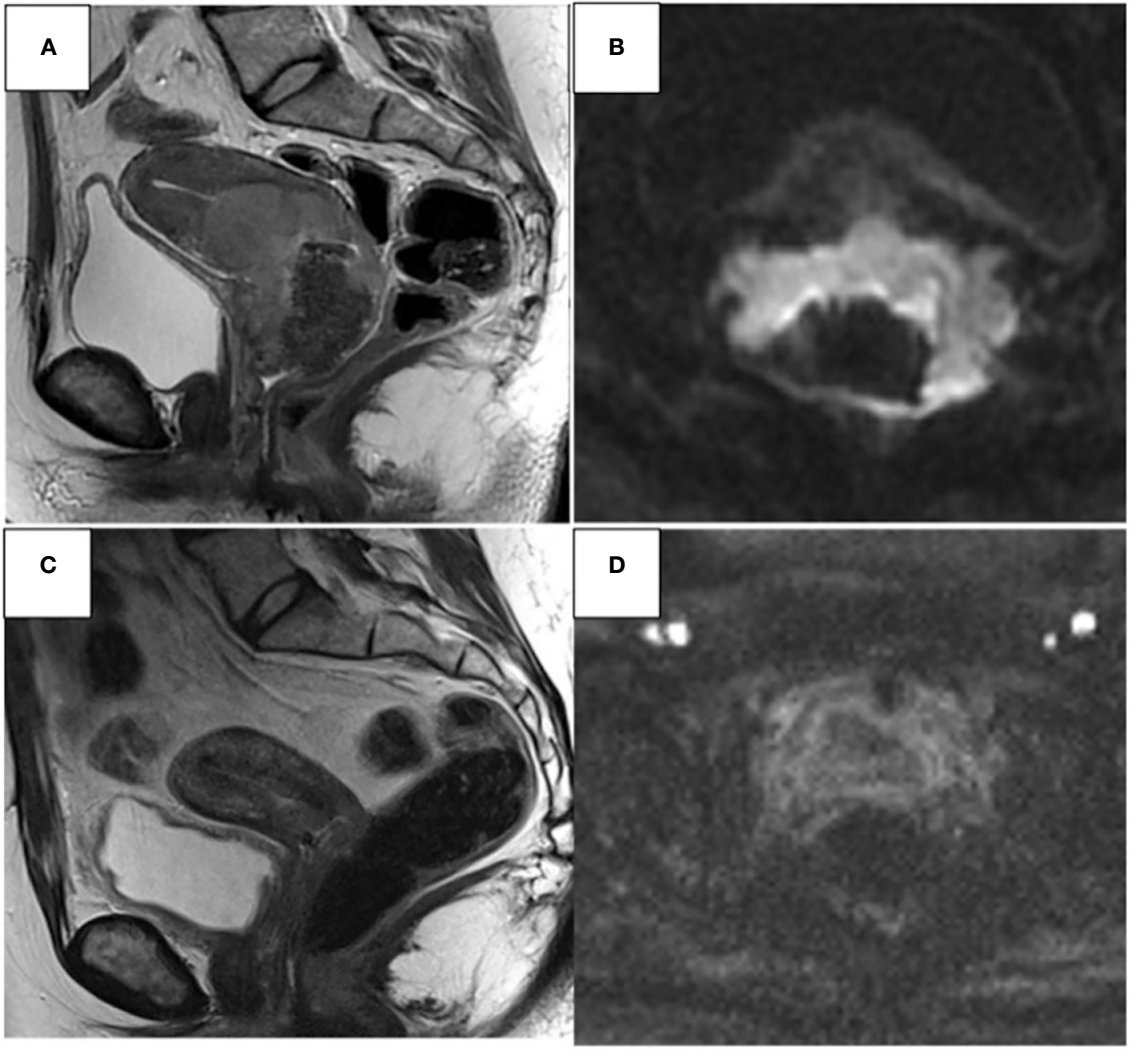
37- year-old woman. Sagittal T2W and axial DWI MR images show CC infiltrating both parameters, the upper 1/3 of the vaginal canal, the uterine body, and the left ureter **(A, B)**. Complete response **(C, D)** significant post-CHT reduction of heteroplastic tissue (around 90%).

**Figure 4:**
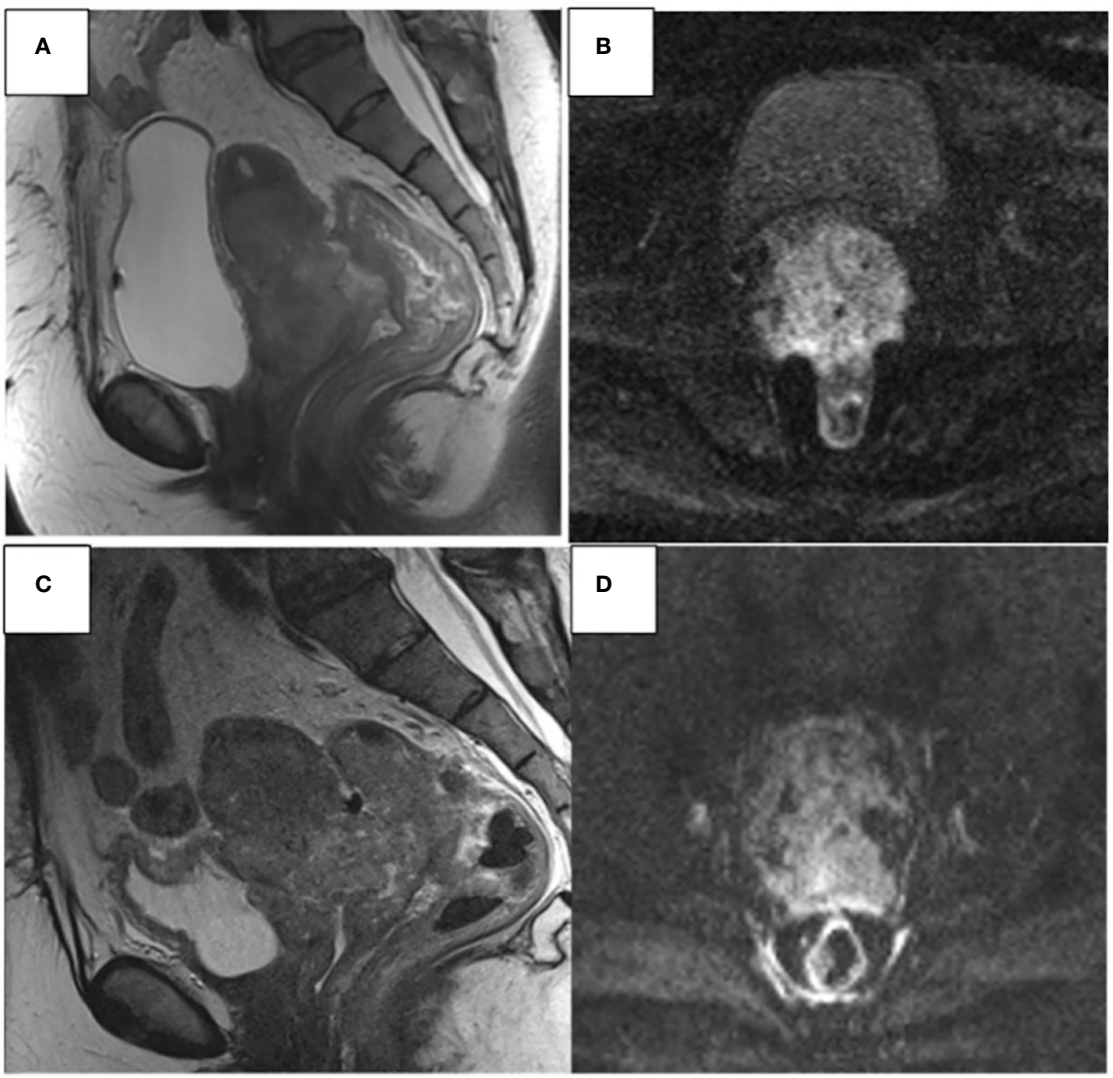
67- year-old woman. Sagittal T2W and axial DWI MR images show an invasive CC **(A, B)**. Progression disease **(C, D)** CC infiltrates the uterine body, lower 1/3 of the vaginal canal, mesorectum, anterior wall of the rectum, and posterior wall of the bladder.

**Figure 5:**
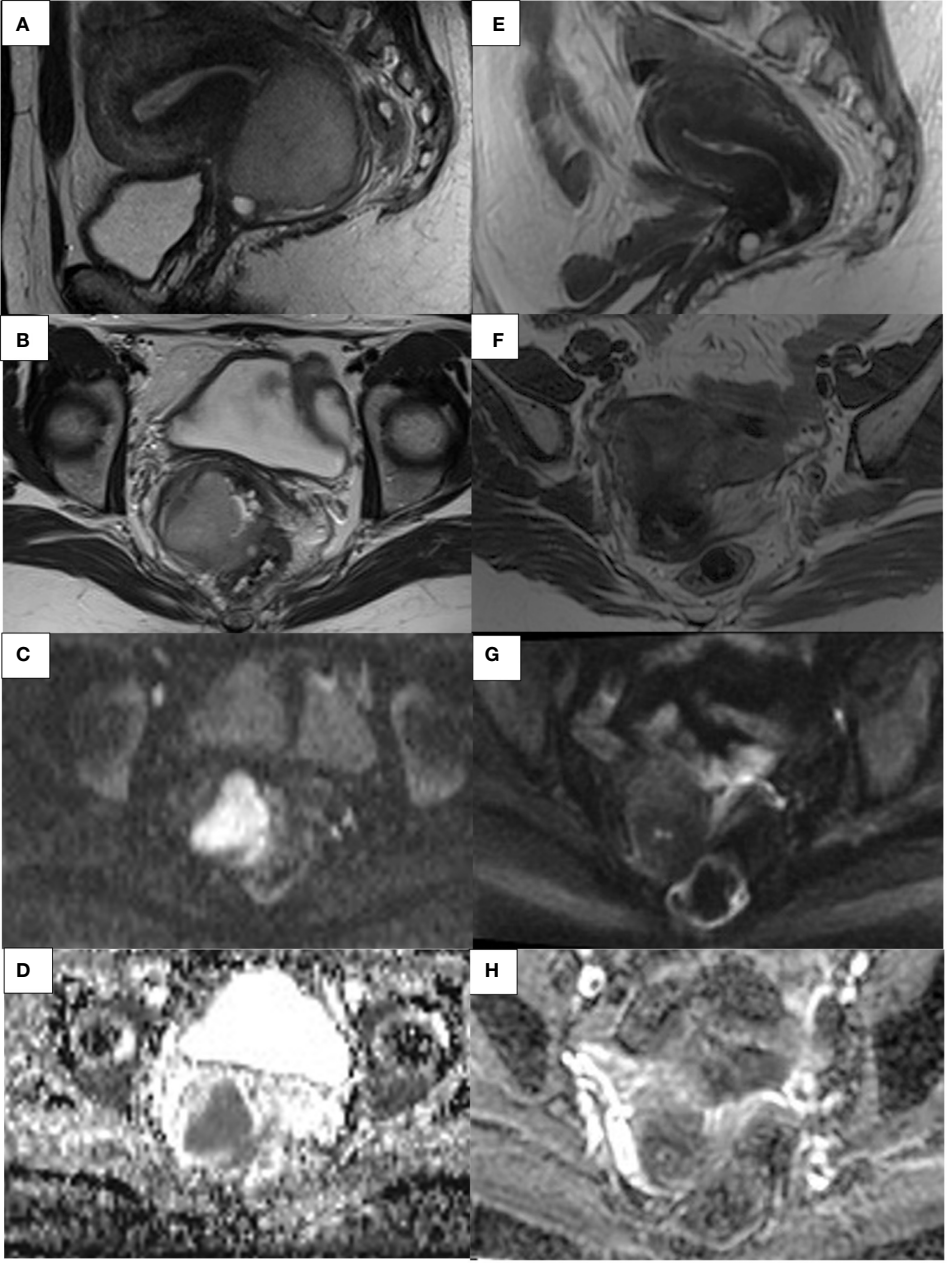
41- year-old woman. Sagittal and axial T2W **(A, B)** and axial DWI MR images with ADC map **(C, D)** show an invasive CC extending to the upper third of the vaginal canal and anterior rectal wall with focal parametrial infiltrations. Sagittal and axial T2W **(E, F)** and axial DWI MRI images with ADC map **(G, H)** shows a complete response after CCRT.

Accurate evaluation of tumor regression after therapy can be used to optimize therapeutic strategy and surgical procedure; to this end MRI is the most reliable imaging modality for patients with LACC due to its high tissue resolution in the pelvis. Tumors treated with chemoradiotherapy (CRT) respond with a decrease in size and signal intensity on MRI. The response may be immediate (3–6 months) or, in larger tumors, delayed (6–9 months) (**Figures 1**–**5**).

#### 4.1.1 T2W-images

Reconstitution of the normal signal hypointensity of stromal ring and homogeneous cervical low signal on T2-W images, is the most important sign of a complete macroscopic response to treatment (9).

Moreover, MRI has the advantages of a multiplanar evaluation of the surrounding structures, providing a clear assessment of the fornix and better definition of the vaginal wall. Normal vaginal vault has a strongly hypointense muscular wall in T2WI, with well-defined and regular contour.

In the first months after therapy, oedema and necrosis caused by CRT may persist for up to 6 months. For this reason the evaluation of the local response can be difficult since the endocervical canal may be enlarged and/or the cervical stroma may show hyperintensity in T2- WI, resulting in a high risk of false positives (30).

In their study Vincens et al. corelated end of treatment MRI results with histopathological findings in patients with CC and found a sensitivity and specificity of 80% and 55% respectively for the detection of residual disease (31).

#### 4.1.2 Diffusion weighted sequences

The recent published ESUR guidelines (2021) for CC provide a central role of DWI sequences which are strictly recommended combined to T2-WI for a correct staging of CC and evaluation recurrence and response after therapy (28).

Traditionally, DWI sequence provides a qualitative evaluation of malignant tumors characterized by high cellular density which causes a restricts water diffusion in the interstitial space. Therefore, the residual disease appears as an area of high signal, especially to high b-value, associated with lower ADC values compared to normal cervical stroma. DWI allows to distinguish the residual tumor from fibrosis, especially in the patients treated with radiotherapy, which on the contrary, presents low signal intensity at high b values and low signal intensity in ADC maps.

A recent multicenter prospective study found that DWI significantly increases the specificity of MRI in detecting local residual tumor compared to T2W imaging alone when assessing cervical cancer response after radiotherapy. In fact, a previous study showed that T2-W sequence alone had a 50% false positive rate (31). Thomeer et al. highlighted that the combination of high intensity on T2-WI and high intensity on DWI was associated with high specificity in the detection of loco-regional residual disease (84%) (32).

Lucas et al. found that combination T2W/DWI had a positive predictive value of 100% and an accuracy of 92.1% for recurrence/residual disease detection, while T2W imaging alone and the combination T2W/DCE-MRI (Dynamic contrast-enhanced MRI) registered values of 93.3% and 80%, respectively (33).

Recently, some studies evaluated how DWI could also provide a quantitative data for CC. Specifically, quantitative analysis of ADC values obtained from a mono-exponential fit to DWI acquired using at least a value of b and b=0 s/mm2 may assume both a prognostic significance and a predictive value for treatment response and local recurrence (34–39).

In particular*, Fu C. et* all advocated that patients treated with neoadjuvant chemotherapy showed an early increased in ADC values before tumor size reduction after 4 weeks of therapy; this value correlates with a reduction in proliferating cell nuclear antigen and cell density suggestive for response to therapy (40).

Somoye et al. demonstrated that median ADC values at mid-treatment were higher in survivors (1.55 × 10-3 mm2/s) than in non-survivors (1.36 × 10-3 mm2/s) with a difference of 14% (41). Some studies, conducted in a large patient population, have pointed out that in cases of complete response the increase of ADC values in early assessment (≤ 2 weeks) is greater than in partial response and therefore the change of ADC value could be a potential biomarker in identifying tumor aggression and treatment-unresponsive disease (40).

##### 4.1.2.1 Intravoxel incoherent motion

Further improvement have been achieved with the introduction of intravoxel incoherent motion (IVIM) which uses a bi-exponential model to fit diffusion signal decay at different b-values (42, 43). IVIM allows to distinguishes the diffusion of water molecules in the extracellular space from capillary micro-perfusion through three quantitative parameters: diffusion “D” (diffusion of water in extracellular space); pseudo-diffusion “D*” (the movement of blood water molecules in the capillary network) and perfusion fraction “fp” (volume percentage of water flowing in the capillaries) (44). Different authors have highlighted correlations between this IVIM parameters and CC regarding: the detection of cervical cancer tissue, the presence of lymph node metastasis and treatment response (45–53). Moreover, recent studies suggested that a IVIM model may also predict the tumor aggressiveness and therapy response showing that D values were significantly higher in good responders patients (*p* = 0.001) and in moderate/high TILs (*p* = 0.018) and that fp showed significantly higher values in squamous cell tumors (*p* = 0.006) (54).

#### 4.1.3 Dynamic contrast enhanced: DCE-MRI

According to the ESUR guidelines, DCE-MRI is not mandatory for local CC staging and its primary application is limited to research setting (55). There is not agreement on the most appropriate use of DCE-MRI and its application remains a challenge. However, some authors have evaluated that DCE may help to detect residual tumor and local recurrences (27, 56). DCE MRI is especially useful in post-treatment imaging because it improves the identification of complete or incomplete response distinguishing between the radiation-induced changes and residual disease (27). From the analysis of DCE time- signal intensity curves, Jalaguier et al. observed that intense enhancement of cervical tissue steeper than the myometrial time- intensity curves in the early stage (type B time- signal intensity curves) is significantly associated with the presence of residual tumor, tumor aggressiveness, incomplete response, worse prognosis, and early recurrence (55).

However, enhancement of the cervix is not specific and is also seen in post-radiotherapy fibrosis, inflammation and necrosis. DCE-MRI in combination with DWI improves the identification of residual/recurrent tumor compared to post-radiotherapy changes. In fact, tumor tissue shows early enhancement, hyperintensity at high b-values and low SI on the ADC map, while fibrosis shows no signal restriction in DWI, has no significant enhancement or shows enhancement in the late phase. Inflammatory changes may show intense enhancement and hyperintensity at high b-values, but have hypersignal in the ADC map (57).

Some studies have evaluated that DCE-MRI during CRT may also has prognostic value. High perfusion before and during CRT suggests increased vascularization and high oxygenation of the lesion and it is related to a better response to treatment and prognosis (56, 58–60) (**Supplementary Table IV**).

### 4.2 PET/CT

In recent years, the role of fluorine-18 fludeoxyglucose (^18^F-FDG) positron emission tomography (PET)/CT in the staging and management of gynecological cancers has been increasing. It is a useful imaging method in the assessment of lymph node and distant metastases in patients with LACC and for assessing response to treatment and disease recurrence (61, 62). Most cervical tumors are 18F-fluorodeoxyglucose (FDG) avid, with exception to adenocarcinomas, which may reveal low FDG uptake. The maximum standardized uptake value (SUV_max_) is currently the most commonly used parameter in ^18^F-FDG PET/CT.

In the context of primary tumor staging, PET/CT plays a valuable role in the evaluation of lymph node metastases. Nodal metastases are frequent in patients with advanced disease (i.e., FIGO stages IIB to IVB) and FDG-PET has been demonstrated to have a high specificity for the detection of nodes in this group of patients). Prospective studies have found sensitivities of 75–100% and specificities of 87–100% (63, 64).

In patients with advanced disease at presentation, PET or PET/CT has been found to alter management in a significant number of patients (65). Sistani et al. reported that the diagnostic sensitivity and specificity of PET/CT to detect residual tumor in patients with LACC were 86% and 95.5% respectively, while the diagnostic sensitivity and specificity to detect distant metastases were 97% and 99%, respectively (61, 62, 66).

Post-treatment FDG-PET/CT is usually performed at 3–6 months after completion of CCRT and it is a valid prognostic biomarker. No FDG uptake indicates a complete metabolic response and consequently a reduced risk of recurrence and excellent survival. Reduced FDG avidity indicates a partial metabolic response and thus a moderately high risk of recurrence and poor survival. Finally, unchanged or new areas of FDG uptake indicate persistent or progressive disease, which is associated with poor survival (33, 67, 68). Early detection of residual tumor is important to establish immediate curative salvage therapy, such as pelvic exenteration or concomitant CCRT (69, 70).

## 5 Complication

After chemoradiotherapy it is important to distinguish between expected changes after radiation therapy in pelvic organs and complications.

Post-radiotherapy complications can also be divided in to acute and chronic complications. The addition of chemotherapy potentiates the acute toxic effects of radiation and also possibly the chronic effects.

Acute toxic effects typically involve the bladder and bowel in the pelvis resulting in radiation cystitis and gastrointestinal symptoms such as colicky abdominal pain, nausea and diarrhea (71). Chronic complications tend to be due to the fibrotic changes in irradiated organs e.g. cervical stenosis, bowel and ureteric strictures. The parametrium soft tissues may also undergo fibrotic changes and appear hypointense. This post-radiation imaging appearance may mimic parametrial invasion thus becoming indistinguishable from the tumor. Vaginal adherents, stenosis, or atrophy are also usually determined. Fistulas are generally late complications of radiotherapy treatment and can also occur as a consequence of a disease recurrence affecting two adjacent organs. For the evaluation of fistulas, MRI is the imaging of choice, which in the sagittal plane are identifiable as hyperintense media in T2-fat suppression sequences and which show impregnation with paramagnetic contrast medium. Other common post-treatment changes are thickening of the bladder and rectal walls, usually associated with diffuse signal hyperintensity in T2-WI, thickening of the utero-sacral ligaments, expansion of the pre-sacral space and diffuse hyperintensity in images. Insufficiency fractures of the sacrum in the post-radiation therapy patient can mimic metastases (7).

## 6 Recurrence of cervical cancer

Recurrence of CC is defined as locoregional re-appearance of the tumor or development of lymph node or distant metastases at least six months after remission of the primary lesion (72). The most frequent locations of recurrence can be classified into central, regional and distant (lymph node or haematogenic metastases).

The central/local site represents the most frequent site of recurrence (30-45%) and includes the vaginal vault, cervix and uterus.

Regional recurrence can be distinguished into anterior (invasion of bladder, urethra), posterior (invasion of anal sphincter, rectum, sigma), lateral (invasion of lateral pelvic wall, iliac vessels, ureters, sciatic nerve, bone) or pelvic lymph nodes (external and internal iliac nodes, obturator nodes).

Distant recurrence includes lymphadenopathies, distinguished into infra-diaphragmatic (para-aortic nodes, inguinal nodes) or supra-diaphragmatic (hilar, mediastinal, axillary, supraclavicular nodes) and distant organ metastases (lungs, adrenal gland, liver, peritoneal carcinomatosis etc.).

The detection of metastatic lymph nodes by MRI and post-contrast CT is based on size and morphological criteria. Lymphadenopathy is characterized by a round shape, irregular margins, internal inhomogeneity and short axis diameter > 10 m; however, recent guidelines suggest short axis of 8 mm as pelvic lymph nodes cut-off (9).

18F-FDG PET/CT is better than the MRI or CT in identifying pathological lymph nodes (18F-FDG PET/CT: sensitivity 84%, specificity 90% and accuracy 87%; MRI: sensitivity 76%, specificity 80% and accuracy 78%; CT: sensitivity 68%, specificity 75% and accuracy 72% (73). 18F-FDG PET/CT also has a high sensitivity (85.7-100%) and specificity (86.7-100%) in the detection of abdominal and extra-abdominal disease (74).

Conversely, MRI is the best imaging technique to detect local recurrence after treatment showing sensitivity and specificity rates of 82-100% and 78-100%, respectively (9).

## 7 Emergent techniques

In the last few decades, ongoing scientific research and technological developments have significantly improved diagnostic accuracy and are approaching to future direction mainly represented by spectroscopy, PET-MRI and radiomics.

### 7.1 Spectroscopy

MR spectroscopy (MRS) is a very sensitive technique that reproduces tissue metabolism and can be used to increase the specificity of non-invasive tissue characterization and prognosis. A recent study analyzed the different lipid profiles in cervical carcinomas with 7 T MRS. It was observed that the 2.1 ppm/1.3 ppm fatty acid ratio could be associated with tumor grade in cervical cancer showing an increase in the amount of unsaturated fatty acids in poorly differentiated tumors. The medium chain of fatty acids becomes less saturated in poorly differentiated tumors (grade III) than in well-differentiated tumours (grade I) or in the normal cervix. Therefore, this ratio may have the potential to characterize tumor grade non-invasively and thus aid clinical diagnosis and management (75). Another recent study demonstrated the feasibility of MRS at 3 T in assessing the correlations between lipid changes in cervical carcinoma and low-prognosis HPV genotypes. MRS demonstrated a significantly elevated fat methyl resonance level at 0.9 ppm in HPV genotypes with a poor prognosis compared to those with a favorable prognosis. Prediction of HPV genotype by MRS may be a useful predictor of the effect of CCRT in patients with advanced cervical cancer, because CCRT is more successful in patients with the poorer prognostic genotype (HPV18- 58) Furthermore, methyl resonance at 0.9 ppm also showed potential in the prediction of persistent tumors after CCRT (76). In addition, methylene resonance at 1.3 ppm has been reported to be more frequently elevated in carcinoma *in situ* (CIN) than in the normal cervix with a sensitivity and specificity of 77% and 94% respectively in predicting the presence of cervical carcinoma (77, 78). MRS, together with morphological and functional MRI, may have the potential to become an integral part of routine MRI examination to add aspects of clinical phenotyping and thus to manage the treatment of cervical cancer patients.

### 7.2 PET/MRI

PET/MRI is an emerging hybrid technique which integrates the high diagnostic accuracy for metastasis and pathological lymph node of PET with the excellent soft tissue differentiation of MRI, strongly required in the local evaluation of gynecological tumors.

Therefore, PET/MRI technique integrates local and distant staging by combining morphological and metabolic information into a single examination that enables response monitoring, surveillance and assessment of recurrence.

Emerging data suggest that for local staging of primary cancer FDG-PET/MRI is equivalent to MRI and superior to FDG-PET/CT; while for lymph node staging it is comparable to FDG-PET/CT (79, 80). Moreover, FDG-PET/MRI is superior to MRI for detecting local recurrence and is highly accurate for identifying lymph nodes and distant metastases (81).

Other studies, highlighted that this hybrid technique has high diagnostic potential in evaluated the suspected recurrence of gynecologic pelvic cancer. Compared with MRI, PET/MRI has been shown superior results in identifying pelvic regional recurrence (82). For example, Sawicki et al. examined 71 women undergoing PET/MRI and MRI for pelvic cancer recurrence and found that PET/MRI correctly identified significantly more patients with cancer recurrence than MRI alone (100 vs. 83.6%) (83).

Therefore, through this new technique we could combine the advantages of two different investigations improving their diagnostic accuracy; moreover, it avoids the ionized radiation necessary for the common PET-CT examination. However, FDG-PET/MRI is poorly used in clinical practice due to limited availability and high cost.

### 7.3 Radiomics

In recent years, radiomics has been assuming a central role and increasing interest in research field; it consists essentially of a cross-disciplinary research area that correlates quantitative data extracted from imaging technique and anatomopathological/clinical information. The ultimate goal of these studies is to develop predictive models that may help identify the most appropriate therapeutic choice for the patient to improve outcome and reduce treatment invasiveness.

Multiple diagnostic techniques are used to stage and evaluate CC: ultrasound, CT, MR, [18F]- fludeoxyglucose (FDG) PET but the latter two are widely considered the most appropriate and therefore object of the main radiomics studies. The main topics of these studies evaluated the correlation with tumor prognostic factors risk facts (histology, parametric invasion and lymph node localizations), response to therapy and prediction of recurrence and distant metastasis.

In particular, in 2017, Tsujikawa et al. affirmed the second-order texture feature extracted by PET/CT imaging discriminating between squamous cell carcinomas (SCCs) and non-squamous cell carcinomas (NSCCs); others studies applied radiomic nomogram from features extracted by MRI and PET imaging to predict the histological grade, lymphovascular space invasion (LVSI) or parametrial invasion (84–86). In addition, other studies have stated that the status of lymph nodes could be predicted by the radiomic pattern developed at first on PET-CT examinations, then on MRI, and ultimately also on ultrasound (US) and CT (87–92).

It is known that around 40% of LACCs undergo disease recurrence. Moreover, in these patients response to therapy is closely related to clinical pathologic prognostic factors but also to phenotypic and genomic features that cannot generally be identified by random sampling or biopsy. Many studies have tried to identify through radiomic analysis these different features to decide pre-operatively the correct therapeutic course. In this context, several studies conducted on both CT, MRI, and PET-CT examination have shown presence of highly predictive model to assess response to therapy (93–99). Similarly, correlations were found between radiomics features extracted from PET and MRI and regional or distant recurrence of disease on which predictive models of recurrence have been based (100). Recently, Lale Umutlu correlated texture features extracted from the innovative hybrid PET-MRI to the presence of N and M-stage resulting that a predictive model may by applying and M-stage prediction was superior compared to N-stage (101).

## 8 Conclusions

In the last decades, great progress has been made in the treatment of patients with LACC (FIGO 2018 stages IB3-IVA). The treatment of choice for LACC is concurrent chemo-radiotherapy, which generally consists of cisplatin-based chemotherapy and external beam radiotherapy followed by brachytherapy. However, the treatment strategy for LACC is still evolving, and there is no consensus on the role of surgery as adjuvant treatment. Imaging plays an important role in the initial and post-treatment evaluation, but also in the planning of radiotherapy allowing to detect residual disease from post-radiotherapy changes, allowing possible salvage therapies. Imaging in combination with chemotherapy and RT increased local disease control, also influencing DFS and OS. Future imaging techniques and scientific research may guide therapeutic management towards more tailored treatment.

## Author contributions

CS and CV have given substantial contributions to manuscript draft; conceptualization, ML and PI; methodology, CS, CV, and ML; investigation, NR, MV and EG; resources, NR and MV; data curation, DM, EG, and GS; writing-original draft preparation, AA, CS, and CV; writing- review and editing, CS, CV, and GS; supervision, ML, PI, and CC; project administration, ML and RP; All authors have read and agreed to be published version of the manuscript.

## Conflict of interest

The authors declare that the research was conducted in the absence of any commercial or financial relationships that could be construed as a potential conflict of interest.

## Publisher’s note

All claims expressed in this article are solely those of the authors and do not necessarily represent those of their affiliated organizations, or those of the publisher, the editors and the reviewers. Any product that may be evaluated in this article, or claim that may be made by its manufacturer, is not guaranteed or endorsed by the publisher.

## Abbreviations

CC, Cervical cancer; LACC, locally advanced cervical cancer; CCRT, Concurrent chemoradiation therapy; RT, Radiotherapy; SCC, Squamous cell carcinoma; LCR, Local Control Rate; OS, Overall Survival; DFS, Disease-Free Survival; NACT, Neoadjuvant chemotherapy; RS, Radical surgery; PFS, Progression-Free Survival; MRI, Magnetic Resonance Imaging; EBRT, External beam radiation therapy; T2WI, T2-weighted imaging; T1WI, T1-weighted imaging; FOV, Field of view; ESUR, European Society of Urogenital Radiology; DWI, Diffusion Weighted Images; FDG-PET, fluorodeoxyglucose-positron emission tomography; LN, Lymph node; NCCN, National Comprehensive Cancer Network; FIGO, International federation of gynecology and obstetrics; CT, Computed Tomography; SI, Signal intensity; ADC, Apparent diffusion coefficient; CRT, Chemoradiotherapy; DCE, Dynamic contrast-enhanced; IVIM, Intravoxel incoherent motion; 18F-FDG, 18F-fluorodeoxyglucose; SUV, Standardized Uptake Value; MRS, MR spectroscopy; CIN, Carcinoma in situ; NSCCs, Non-squamous cell carcinomas; LVSI, Lymphovascular space invasion; US, Ultrasound.
