# Supplementary material for: Post treatment imaging in patients with local advanced cervical carcinoma
Source: Front Oncol. 2022 Oct 27;12:1003930. doi: 10.3389/fonc.2022.1003930 (PMC9710522; doi:10.3389/fonc.2022.1003930)
Supplement: Supplementary file 1 [file Table_1.docx]

Table I:

| **II: carcinoma invades beyond uterus** | | **IV: Extension involved bladder or**  **rectum** |
| --- | --- | --- |
| IIA: Invasive carcinoma without parametrial invasion  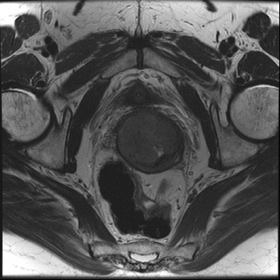 | IIB: Invasive carcinoma  + parametrial invasion  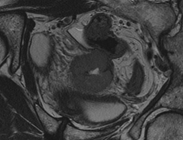 | IVA: Spread adjacent organs  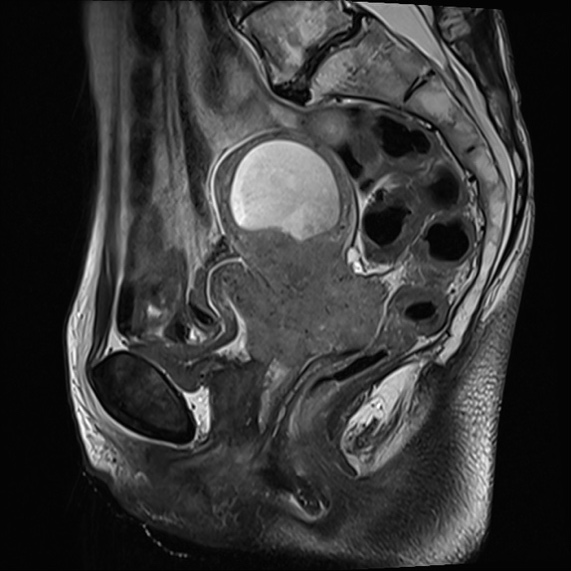 |
| **III: Invasion of the lower 1/3 of the vagina/hydronephrosis/ nonfunctioning**  **Kidney/pelvis wall/pelvic-paraaortic nodes** | | |
| IIIA : Lower 1/3 of the  vagina, with no extension to the pelvic wall  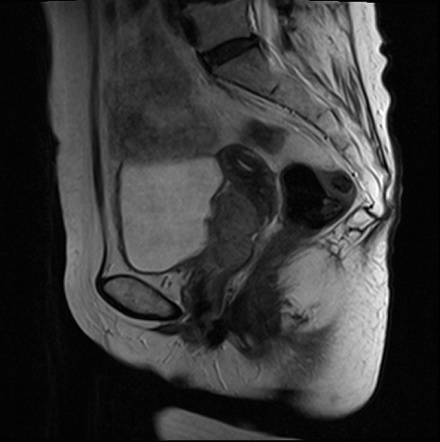 | 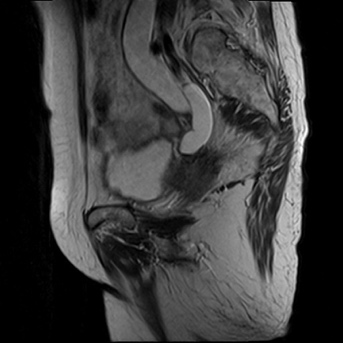IIIB : Extension to the pelvic wall and or causes hydronephrosis or nonfunctioning kidney | IIIC : Involvement of pelvic and/or para-aortic lymph nodes  *IIIC1:Pelvic nodes metastasis only*  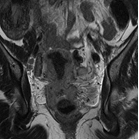*IIIC2 :Para-aortic nodes metastasis* |

Table II:

| **Concurrent chemoradiotherapy (CCRT) vs RT** | | | | |
| --- | --- | --- | --- | --- |
| **Author** | **Year** | **N** | **Stage** | **Results** |
| Datta | 2017 | 2445 | IIB-IVA | CRT improves outcomes, LCR and OS |
| Claire Vale | 2008 | 3452 | IB-IVA | CCRT 6% improvement in 5-year survival |
| **CCRT followed by surgery versus CCRT** | | | | |
| **Author** | **Year** | **N** | **Stage** | **Results** |
| Lèguevaque | 2010 | 111 | IB1-IVA | CCRT + RS improve DFS but not OS |
| Fanfani | 2016 | 150 | IIIA-IIIB | No significant differences of DFS and OS |
| Yoshida | 2019 | 126 | IB2-IIB | CCRT+ RS improve DFS and OS, and reduced pelvic recurrence |
| Kim G.G. Van Kol | 2021 | 220 | IB-IIIB | Histological confirmation required before salvage surgery |
| **NACT followed by surgery versus CCRT** | | | | |
| **Author** | **Year** | **N** | **Stage** | **Results** |
| Benedetti Panici | 2002 | 441 | IB2-III | NACT + RS are a valid option with acceptable survival and mild surgical complications |
| Gupta | 2018 | 635 | IB2-IIB | NACT+ RS has lower DFS than CCRT  Not significant difference in OS |
| Zhao | 2019 | 2158 | IB2-IIB | Demonstrated no differences in terms of OS, PFS, local or distant recurrences |

|  | TR/TE (ms) | FOV (mm) | NEX | Matrix Size | Slice Thickness (mm) | Intersection gap (mm) | B values (s/mm2) | FA (°) | Temporal resolution (s) |
| --- | --- | --- | --- | --- | --- | --- | --- | --- | --- |
| Sagittal FSE T2WI | 3411/121 | 320x320 | 2 | 320x224 | 4 | 1 | / | / | / |
| Para-axial, Para-coronal FSE T2WI | 5089/127 | 240x240 | 2 | 320x224 | 4 | 1 | / | / | / |
| Para-axial FSE T1WI (w/wo FS) | 400/ | 240x240 | 2 |  | 4 | 1 | / | / | / |
| Para-axial DWI | 2000/57 | 240x240 | 2 | 160x80 | 3,5 | 0 | 0-500-1000 | / | / |
| Axial DWI | 2000/57 | 240x240 | 2 | 160x80 | 3,5 | 0 | 0- 500-1000 | / | / |
| Axial FSE T2WI | 5000/125 | 310x310 | 2 | 320x244 | 4 | 1 | / | / | / |

Table III:

Table IV:

| Peals and pitfall of MRI imaging for the evaluation of treatment response in the LACC | |
| --- | --- |
| T2 | PEARLS  Evaluation of immediate (3–6 months) or delayed (6–9 months) response represented by normal signal hypointensity of stromal ring and homogeneous cervical low signal;  Evaluation of fibrotic scar tissue whose T2WI signal is lower than the normal muscular tissue;  Better definition of the fornix and the vaginal wall: strongly hypointense wall.  PITFALLS  Small recurrence or residual may be undetectable only with T2;  Post-radiotherapy oedema may hide residual tumor on T2. |
| DWI | PEARLS  Increase the accuracy of T2 sequences in the detection of small tumors;  Increase the accuracy of T2 sequences in the evaluation of residual tumor after radiotherapy;  Identifying tumor aggression and treatment-unresponsive disease: increase in the value of ADC in the early assessment (≤ 2 weeks) is greater in patients with complete response than those with partial response.  PITFALLS  More affected by artifacts. |
| DCE-MRI | PEARLS  Evaluation of response after chemo-radiotherapy:   - improve the detection of small tumors characterized by large necrotic component; - persistent enhancement in the post-surgical bed probably indicates residual disease.   Prognostic value:   - High perfusion before and during CRT suggests increased vascularisation and high oxygenation of the lesion and it is related to a better response to treatment and prognosis   PITFALLS  Highly variable timing of post-treatment MRI limits conclusions;  enhancement of the cervix is non-specific and is seen also in cases of postirradiation fibrosis, inflammation and radiation necrosis. |
